# Effects of Physical Forms of Total Mixed Rations on Intake, Weaning Age, Growth Performance, and Blood Metabolites of Crossbred Dairy Calves

**DOI:** 10.3390/ani9080495

**Published:** 2019-07-27

**Authors:** Zahid Iqbal, Muhammad A. Rashid, Talat N. Pasha, Jalees Ahmed

**Affiliations:** 1 Department of Animal Nutrition, University of Veterinary and Animal Sciences, Lahore-54000, Pakistan; 2 Department of Livestock Production, University of Veterinary and Animal Sciences, Lahore-54000, Pakistan

**Keywords:** calf, oat hay, pelleted, performance

## Abstract

**Simple Summary:**

Weaning the calf earlier has been found to reduce the milk feeding and labor cost through higher solid feed intake and early rumen development. Forages, when included in calf starter diet, have been found to be beneficial in this regard. This experiment was planned to evaluate the effects of adding 15% oat hay to calf starter and feeding the same as pelleted or un-pelleted total mix ration. Three groups of calves were given four liters of milk daily during the first week and then six liters daily with free access to water and the respective starter diet. The quantity of milk was gradually reduced until the calf started consuming 800 grams of starter for three days. Feed intake, health scores, and shed temperature/humidity were recorded daily. Body weight, body measurements, and blood sample collections were carried out weekly. Feed intake and body measurement gains were similar in all groups. Blood metabolite concentrations and rumen development parameters were also not affected. Average daily weight gain and feed efficiency were numerically higher for calves given pelleted total mix ration followed by unpelleted total mix ration and only concentrate starter feed. On average, calves fed pelleted total mix ration were weaned 5.4 days earlier than those fed only concentrate. No negative effect was observed on any health or growth parameter with addition of oat hay. Expenditure per kg of weight gain was numerically lowest in the pellet fed group, followed by hay mix starter and only starter fed calves. Oat hay may be safely included in calf starter ration as pelleted mixture with economic benefits.

**Abstract:**

This study evaluated the effects of hay-based total mix ration (TMR) in pelleted or unpelleted form on intake, weaning age, performance parameters, blood metabolites, and cost-effectiveness in crossbred dairy calves during the preweaning period under hot climatic conditions. Thirty calves (4 ± 1 days (d) old), were assigned to one of the three dietary treatments in a randomized complete block design. Treatments assigned were: (1) conventional calf starter (CCS); without hay (2) TMR containing 85% calf starter and 15% chopped oat hay (OH) and (3) pelleted TMR (PTMR) containing 85% calf starter and 15% OH. Colostrum-fed calves were offered four liters (L) of whole milk each, during the first week, followed by six L daily. Calves were weaned off milk gradually on the basis of targeted starter intake of 200, 600, and 800 grams, after which they were considered weaned. Environmental temperature, feed intake, and health scores were recorded daily. The calves experienced heat stress with an average temperature-humidity index (THI) of 80.06 ± 3.42. Results revealed that average daily dry matter intake (DMI), average daily gain (ADG), and feed efficiency (FE) were not different (*P* > 0.05) among the treatments. Changes in body measurements, rumen development parameters, and blood metabolites were also not affected by the treatments (*P* > 0.05). Average weaning age in PTMR was 5.4 d earlier as compared to CCS. Feed cost per unit gain tended to be lower for PTMR compared with CCS-fed (86%) calves. Feeding pelleted TMR containing 15% OH tended to reduce the weaning age and feed cost per unit gain without affecting growth performance, intake, FE, and health parameters in crossbred calves under heat stress conditions.

## 1. Introduction

Calves, being the replacement herd of a dairy farm, require efficient nutritional management which should not only improve their health and growth but also reduce the expenditure to be incurred by their rearing. Feeding young calves is a high cost of heifer rearing and therefore nutritionists are investigating the strategies that can reduce the charges of this phase. The nature and form of solid feed and its quantity consumed affects types of microbes and pace of rumen development [1]. Typically, young calves are fed starter feeds high in grain to allow earlier development of rumen [2,3]. However, free choice calf starter feeding during the preweaning period have been shown to lower rumen pH [1], decrease ruminal motility, and cause hyperkeratinization, leading to rumen papillae clumping [4].

The amount and type of roughage to be offered to the young calf are still unclear [2]. To date, studies involving the use of roughages in calf diet have given variable results. The addition of forages to the diet has been found to result in lower DMI and weight gain, less β-hydroxybutyrate (BHBA) [5,6], and consequently delayed rumen development [7,8]. Other research has suggested that inclusion of dry roughage to the calf diet stimulates the muscular layer of the rumen [7], promotes rumination [9,10], and reduces ruminal plaque formation [11].

One possible way to increase the DMI of forage-based starter is to reduce the particle size. Earlier work in calves by Coverdale et al. [12] reported that 15% inclusion of consistent 8–19 mm particle size hay improved intake, ADG, and feed efficiency in calves. Similarly, Castells et al. [13] reported that provision of free-choice chopped forage to milk-fed young calves resulted in higher feed intake and better performance without any negative impact on digestibility. Ackeren et al. [14] documented that early feeding dry TMR containing 15% alfalfa hay and weaning at 8 weeks improved the weight gain at 15 weeks as compared to those fed component feeds.

Another way of improving the intake of forage-based diets in young calves could be the inclusion of chopped/ground fiber sources as a TMR. Greater intake, higher neutral detergent fiber (NDF)/acid detergent fiber (ADF) digestibility and a trend of higher gain to feed ratio was achieved in calves fed high fiber course mash TMR than those fed fine ground [15] or pelleted TMR [16]. On the other hand, higher weight gain was observed when TMR pellets consisting of finely ground straw and starter were fed until 8 weeks after weaning [17]. Recently, Suarez-Mena et al. [18] evaluated the use of low quality straw source with varying chop length. The study concluded that varying particles size of straw included at 5% had a minimal effect on ruminal fermentation indices, rumen pH, as well as rumen morphometry. Most recently, Kehoe et al. [19] documented that calves can be fed on TMR containing 50% starter and 50% corn silage without any detrimental effects on ADG, health, and intestinal morphometry.

In adult cattle and sheep, forage particles >1.18 mm leaving the rumen is considered as a threshold [20], and therefore can suffice as a stable rumen environment and provide a necessary abrasive factor for smoother papillae development [21].

Inclusion of forages, in preweaned diets of young calves at >10% of the ration dry matter (DM) is not encouraged due to possible depression in the intake. However, recent work in small ruminants fattening showed that higher levels, up to 25% of finely ground straw at 2 mm in light fattening lamb, increased intake, growth performance, and reduced the incidence of acidosis [22]. To date, limited published information is available on the comparative evaluation of conventional and pelleted TMR in milk-fed calves. Therefore, we hypothesized that straw-based pelleted TMR can reduce the weaning age through improved intake without affecting the growth performance of dairy calves. The objectives of this study were to determine the effects of physical form (conventional vs. pelleted) of complete feeds containing 15% fine ground OH on intake, weaning age, growth performance, blood metabolites, and rumen morphometry of dairy calves.

## 2. Materials and Methods

The experiment was approved by Animal Care and Use Committee (ACUC) (approval code for the submitted manuscript is DR/727 dated July 19-2017) the University of Veterinary and Animal Sciences (UVAS) Lahore and was carried out at Military Farm Renala, Okara Pakistan from May to August.

### 2.1. Experimental Design

Thirty newly born, crossbred (Frisian × Sahiwal) cow calves were blocked by sex and assigned to three dietary treatments with *n* = 10 (five male and five female) calves in each treatment. Treatments were: (1) conventional calf starter (CCS) without hay (2) TMR containing 85% calf starter and 15% chopped OH with a chop length of 1.5 cm (3) pelleted TMR (PTMR) containing 85% calf starter and 15% OH. The hay was ground in a hammer mill so that it could pass through a sieve of 2 mm before uniformly mixing with grain starter for steam pelleting. Pellet size was 4 × 18 mm made at a temperature of 65–70 °C. All the experimental diets were isonitrogenous. Ingredient composition of treatments and their nutritive profile on DM basis is given at Table 1.

### 2.2. Feeding, Weaning, and Husbandry

The duration of the experiment was 84 d. The calves were enrolled in study after ad libitum feeding of colostrum for 3 d. Calves were housed in individual steel pens (2.13 × 1.22 × 1.17 m) bedded with rubber mats. The pens were placed in two rows under a naturally ventilated open-sided shed. Electric overhead fans installed in the shed were used for cooling. During week 1, calves were fed whole milk, 4 L/d in two equal feedings at 0830 hours (h) and 1800 h. However, from week 2 to until the start of weaning, calves were fed 6 L/d, in two equal feedings. The milk offered to the calves was diverted from saleable production of the farm having at an average 11.9% total solids, 4.08% fat, and 2.89% protein. Calves were weaned off milk using the starter intake-based weaning (SIW) protocol described by De Passillé and Rushen [23]. Briefly; daily milk allowances were adjusted on the basis of three-step targeted starter intake of 200, 600, and 800 g of starter intake for three consecutive days. At intermediate steps 1 and 2, milk allowance was reduced by 25% and fed twice daily. When the calf reached 50% reduction in milk allowance, milk feeding was carried out in the morning only. For all calves, the weaning was completed when individual calf starter intake reached to 800 g/d for three consecutive days. Milk feeding was carried out using steel buckets, and feeding utensils were washed using a sanitizer after every feeding. Calf starter and water were offered ad libitum individually in plastic buckets and measured on a daily basis.

### 2.3. Performance, Health, Sampling, and Analysis

Health parameters including fecal consistency, respiratory problems, and general appearance were recorded on daily basis on a scoring system from 1–5, 1 being normal and 5 being grave, as described by Heinrichs et al. [24]. Animals with fecal score >3 and temperature >39.5 °C were treated according to farm protocols. Calves with scours were given oral rehydrant for three consecutive days or until the signs of sickness disappeared. Samples of starter and TMR feed were collected fortnightly, dried at 55 °C in a forced air oven and ground to 1 mm using a Willy mill. Samples were used to determine: DM contents (using hot air oven at 105 °C for 3 h), crude protein (CP) and crude fat [25]. Samples were ashed by igniting the samples at 550 °C for 4 h in a muffle furnace. An α-amylase + sodium sulfite treated filtration methods were used for sequential determination of the NDF; whereas, an sulfuric acid + cetyltrimethylammonium bromide treated filtration methods were used for the determination of ADF) by using an Ankom 2000 fiber analyzer (Macedon, NY, USA) [25,26].

### 2.4. Morphometry

Four male calves from each treatment were slaughtered with Islamic method in Butchery of Meat Science and Technology Department (UVAS, Lahore). After deskinning, rumen and reticulum were tied at pyloric and esophageal ends and removed by cutting between two knots. Similarly, remaining digestive and visceral organs were also taken out and weighed. Ruminal and intestinal contents were removed, organs washed with cold water, and reweighed. After visual examination rumen tissue samples were dissected as per procedures given by Lesmeister et al. [27] and preserved in 30% formaldehyde solution for subsequent analysis. Morphometric analysis was carried out under Labomed Stereo Microscope using software Labomed Pixle pro.

### 2.5. Blood Sampling and Analysis

Weekly blood samples were collected, four h post morning feeding, using EDTA coated and non-EDTA vacutainers from jugular vein to harvest plasma and serum, respectively. Harvested plasma and serum samples were divided into aliquots and stored at −20 °C until further analysis. Plasma glucose and BUN were determined using colorimetric kits (catalogue # 11538: BioSystems; catalogue # 11537: BioSystems). Plasma BHBA concentration was measured using a colorimetric kit (Catalogue # H7587-58: POINTE SCIENTIFIC, INC).

### 2.6. Body Weight and Body Measurements

Body weight (BW) was measured at the start of experiment and then on a weekly basis using a digital weighing scale. Body measurements were carried out at the start and then on a weekly basis. Wither height (WH) was measured with measuring stick from base of fore foot to the highest point of the wither, heart girth (HG) was measured as a circumference by wrapping a tape around chest region just behind elbow, hip height (HH) was measured from base of the hind foot up to the hook bone and body length (BL) was measured as distance from point of shoulder to point of rump [28,29].

### 2.7. Cost of Feeding

Cost of feeding per kg gain was calculated by dividing total amount expended on milk and starter feed consumed by total weight gained in kg by the calf at weaning.

### 2.8. Feed Efficiency (Entire Experiment)

Feed efficiency was calculated by dividing kg of weight gained by DMI in kg.

### 2.9. Temperature Humidity Index (THI) and Wind Velocity

The temperature and humidity were recorded twice daily by using TH meter. Height of the TH meter from ground was 2.5 m. The temperature humidity index was calculated using the formula [30] given below;

THI = (0.8 × dry bulb temperature) + (% relative humidity/100) × (dry bulb temperature − 14.4) + 46.4

Wind velocity was measured daily at 1400 h by using smart sensor electronic anemometer (AR816; Guangdong, China).

### 2.10. Statistical Analysis

Data were analyzed as a randomized complete block design using mixed procedures of SAS [31]. Initial body weight was used as a covariate for milk and starter intake, DMI, BW gain, ADG, and feed efficiency. Initial measurements were used as a covariate for analysis of final measurements and gain in WH, HG, HH, and BL. Starter DMI was used as a covariate for rumen development measures and organ weights. Effect of sex was analyzed as a fixed factor, found nonsignificant (*P* > 0.05), and therefore removed from the model. Data measured over time was summarized weekly for individual calf and analyzed with repeated measures ANOVA. The model included fixed effects of treatment, week, and week × treatment interaction and calf as a random factor. Difference among means was considered significant at *P* ≤ 0.05 and trend reported at *P* < 0.10. Mean values have been expressed as least square means. All the data were subjected to nonparametric SNK test to determine the normal distribution.

## 3. Results

### 3.1. Meteorological Parameters

Averages of peak daily temperature, wind velocity, humidity, and THI are presented in Table 2. The values are significantly higher than the thermoneutral zone determined for neonatal calves by previous researchers [32,33].

### 3.2. Feed Intake

**Figure 1:**
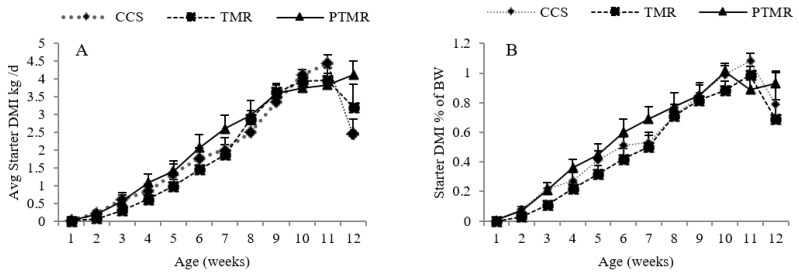
Weekly least square means of starter DMI kg/d (**A**) and starter DMI % of BW (**B**) of crossbred dairy calves fed conventional calf starter (CCS) and oat hay as TMR or in pelleted form (PTMR).

Least square means of feed intake are presented in Table 3. Total milk intake at weaning was numerically less in PTMR than the other two treatments. Average starter DMI kg/d and starter DMI % of BW (Figure 1A,B) were numerically higher for PTMR as compared with CCS and TMR.

### 3.3. Body Weight, Average Daily Gain, Feed Efficiency, and Body Measurements

Least square means of BW, ADG, and FE are presented in Table 3. Initial and final BW were not different among treatments (*P* > 0.10). Gain in BW was similar among treatments at weaning (*P* > 0.10). Calves in all the groups gained weight over the week with advancing age (*P* < 0.05). Average daily gain (ADG) and gain to feed ratio/FE were numerically higher for PTMR followed by TMR and CCS. Least square means of body measurement gains are shown in Table 4. Initial and final measurements of body conformation were not different among treatments. Gain in all body measurements was observed with age (*P* < 0.01). However, gain in wither height, heart girth, hip height, and body length were similar among treatments at weaning (*P* > 0.05). A tendency (*P* = 0.06) in treatment × week interaction was observed for body length gain during week 6 and 12 when this measure was higher for PTMR calves compared with CCS and TMR.

### 3.4. Weaning Age and Cost per Kg

Weaning age tended to be lower in PTMR (*P* = 0.07) as compared to the CCS and TMR calves. Average feeding cost per kg gain tended to be lower (*P* < 0.10) in PTMR versus (vs.) CCS. Mean values for weaning age and cost per kg gain are also shown in Table 3.

### 3.5. Blood Metabolites

Mean values for blood metabolites of calves are given in Table 5. Blood urea nitrogen concentration was similar among treatments. Mean BHBA values were not different among treatments; however, concentration increased in all groups with age. Mean plasma glucose concentration was also not different; however, it decreased with age in all treatments.

### 3.6. Organ Weights and Morphometry

Means values of filled and unfilled organ weights and rumen morphometry are given in Table 6 and Table 7. Body weights with or without rumen ingesta and empty body weight (EBW) % of BW as well as carcass weight were similar (*P* > 0.10) for the treatments. Rumen and reticulum weights with and without ingesta as well as rumen weight % of EBW were also not different (*P* > 0.10). Filled and unfilled weights of omasum, abomasum and intestine, omasum % of EBW, as well as weights of liver, spleen, and kidneys were also not affected (*P* > 0.10) by the treatments. Rumen development parameters that is papillae length, papillae width, rumen wall thickness, and papillae concentration were also not found affected (*P* > 0.10) by the treatments.

### 3.7. Health Parameters

Two calves were removed from the study (Treatment 2) during the initial week; one due to a skin problem and a second due to a limb injury. All other calves remained healthy and no serious problem was noticed. Fecal score, respiratory score, and general appearance were not affected by the treatments (*P* > 0.05). Fecal score remained higher during the initial week but decreased later as milk allowance was reduced. Least square means are given in Table 8.

## 4. Discussion

### 4.1. Feed Intake

As expected starter intake increased in all the groups with age which shows rumen development, enhanced activity, and more capacity due to gene–nutrient interaction [34]. Similar starter DMI in all treatments is in agreement with a number of previous studies [11,12,35]. This indicates that inclusion of OH did not affect starter intake as documented by Hill et al. [6] with the addition of grass hay.

Khan et al. [28] found higher DMI in hay and starter fed calves from six to ten weeks of age. The higher DMI in their case may be attributed to decrease in milk allowance at week 5, because calves were given milk allowance at 20 % of their BW. They consumed minuscule (<100 g/d) quantities of solid feed until milk allowance was reduced to half. This step-down milk feeding urged calves to consume more solid feed, whereas, in our experiment milk allowance was reduced on the basis of starter feed intake. Moreover, they also recorded DMI for two more weeks after weaning. The results are also in contrast with Castells et al. [13] who found higher starter intake as well as total DMI in calves fed hay. The possible reason could be ad libitum feeding of hay separately and weaning at a fixed time which might have led to higher gut fill which was not determined by the authors. In our study, weaning was based on solid feed intake and hay was mixed with starter in a fixed percentage. Hill et al. [6], in one of their experiments, found a decline in starter intake with an increase in inclusion level of hay in the diet but in that study calves used were initially 58 to 60 d of age and had also been used previously in another trial. However, in their third experiment, preweaning starter intake was not affected by addition of 5% hay.

### 4.2. Performance

Our results indicated no statistical difference in performance parameters of preweaned calves fed CCS, TMR, and PTMR. However, numerically, the calves fed PTMR consumed 7.14% more starter DMI/d, gained 4.38% more weight, 5.56% higher ADG, and had 8.06% better gain to feed ratio as compared to calves fed CCS. Calves fed PTMR also tended to have lower weaning age. The addition of OH in the diet of young calves creates a better rumen environment and does not increase gut fill as also reported by Castells et al. [36]. Since calves in this group got weaned earlier, cost per kg gain at weaning was 14% lower than CCS on live weight basis. These findings are in line with Coverdale et al. [12] who also found similar results prior to weaning in a series of two experiments conducted on Holstein bull calves (experiment 1) and Holstein, Jersey, Ayrshire, and Brown Swiss calves (experiment 2). Coverdale et al. [12] compared feeding of coarsely and finely ground starter with addition of 7.5% and 15% grass hay and found numerically higher values for BW, ADG, and FE prior to weaning but the difference was not significant. Moreover, starter intake and DMI were not affected by the addition of hay prior to weaning. However, they continued the experiment after weaning on the same diet and found significant improvement. There was no considerable difference in weaning age of treatment groups in our experiment, which is also in line with Coverdale et al. [12].

Khan et al. also [28] found similar BW in calves fed forage or non forage-based diets; however, the authors found improvement in total starter DMI after five weeks of age when quantity of milk was reduced. In contrast, Hill et al. [6] found depression in ADG, starter intake, and FE in calves fed starter blended with 15% hay as compared to those fed 5% hay or no hay during the pre and postweaning period. Negative effects observed in their study might be due to different NDF and ADF content. They used cotton seed hulls (NDF 79.2%) and mixed type of hay (NDF 46.6%) for feeding and used straw bedding which might have contributed to lower digestibility. The NDF content of CCS in our case was 21.78% and that in TMR and PTMR was 28.75%. Suárez et al. [11] also found no effect on DMI and ADG during the preweaning period by substituting a part of the concentrate with roughages. The results of our experiments also partly substantiate the findings of Castells et al. [13], who compared the effect of using various roughage sources and found similar FE in calves fed chopped hay as compared to conventional grain starter. However, they found significant improvement in BW, ADG, starter intake, and DMI % BW by offering OH ad libitum, giving free choice to calves fed hay up to 10% of total starter diet. We did not find such increase in these parameters with use of fixed 15% OH. Similar to this study, Jahani-Moghadam et al. [35] found no considerable effect of feeding hay to calves on feed intake, ADG, and FE. They also reported no advantage of feeding pelleted vs. chopped hay. According to most of the recent research results, addition of forage to calves’ diet during the milk feeding stage raises rumen pH, increases slivery secretion, and maintains healthy gut function without compromising feed intake and body weight gain [37]. Numerical improvements were also observed in structural gains such as WH, HG, HH, and BL in calves fed TMR and PTMR as compared to CCS. Structural measurements determined in our experiment commensurate with BW gain and feed intake. Addition of forage to calves’ diet can improve their performance, but the level of effect depends upon its source, amount, and physical form being fed [38].

The strategy of feeding pelleted TMR to preweaning calves is practical for farmers and can economize heifer rearing by reducing cost on account of milk/milk replacer and labor. To the best of the authors’ knowledge, this is the first study of this nature carried out on crossbred calves under hot summer conditions and further studies are required with varying inclusion levels, types of hay, and weaning targets.

### 4.3. Blood Metabolites

Unaffected plasma BHBA concentration for the three treatments before weaning is in agreement with previous researchers [12,28,39] who evaluated effects of feeding hay to young calves. Similar BHBA values in all the three treatments indicate that addition of OH in starter diet duly mixed as TMR or processed in pelleted form does not affect function of the rumen epithelium. Plasma BHBA concentration possibly reflects ruminal activity for metabolism of butyrate [40], which was not compromised by addition of forage source. Preweaning values of BHBA concentration found in this experiment are higher than Coverdale et al. [12] and lower than Quigley, et al. [41]. In a former experiment, calves were weaned earlier than ours, that is why their entire trial values (postweaning) were closer to current study, whereas, in the later case, values varied due to the greater age of calves and blood collection time after feeding. Moreover, a concurrently higher (95 mol/100 mol) proportion of acetate in rumen volatile fatty acids (VFA) was observed in the experiment carried out by Quigley et al. [41]. The plasma BHBA concentration increased with the age of calves as found by other research workers [28] due to higher solid feed intake [42] and resultant conversion of butyrate to BHBA [28]. Blood glucose and blood urea nitrogen (BUN) were not different among treatments, which is in line with previous authors [35,39]. Blood glucose values decreased with age as milk allowance was gradually reduced due to shift in primary source of energy from glucose to volatile fatty acids, which is also in line with other studies [35,43,44,45].

### 4.4. Organ Weights and Morphometry

The organ weight results of this study and rumen development parameters are generally in line with previous workers who also found similar values in starter + hay fed calves in comparison to starter fed calves [28]. However, rumen and reticulum weight with and without ingesta were found to be heavier in hay fed calves as compared to only starter fed calves by Khan et al. [28], which is in contrast to our findings. This may be because they weaned the calves at the 56th day of age but killed them for morphometry study at the 70th day of the experiment. It means the calves consumed hay for two more weeks after weaning and as reported, consumed more DM during 6 to 10 weeks of age. Whereas, in our case, the calves were slaughtered soon after weaning. In another study, Castells et al. [36] also found statistically similar rumen reticulum % of EBW in hay (AH & OH) fed calves vs. starter fed calves; however, they [36] found higher abomasum % of EBW in hay fed calves, which was not observed in our case. It again may be due to free-choice feeding of hay during preweaning as well as three weeks after weaning as the male calves were sacrificed at 71 days of age.

### 4.5. Cost per kg

In addition to savings due to early weaning and numerically better gain to feed ratio, the cost per kg of TMR and PTMR was also lower. Grains and meals are the most expensive feed ingredients in developing countries. Oat is a winter crop commonly grown by farmers for fodder which can be easily converted to hay and is known for its high palatability in calves. Its inclusion in calf starter can considerably reduce the cost of starter feed for calves. Addition of hay to calves ration lowered its cost without any negative impact on performance [35]. Moreover, easy handling and transportation of pelleted TMR can help commercial producers to adapt and manufacture specialized forage-based TMR for farmers.

## 5. Conclusions

Feeding pelleted TMR containing 15% oat hay tended to reduce the weaning age by five days and feed cost per unit gain by 14%, as compared to conventional calf starter, without affecting growth performance, intake, FE, and health parameters in crossbred calves under heat stress conditions. The calves used in this experiment were *Bos indicus* influenced and the results may be different in dairy breeds of European ancestry. Further studies are warranted to determine the optimal levels and particle size of hay in pelleted TMR for dairy calves.

## Figures and Tables

**Table 1:** Ingredient composition of starter feed and total mixed rations used in the preweaning experiment.

Ingredients	CCS (%)	Hay Based TMR (%)
Ground maize	40.0	37.0
Oat hay	-	15.0
Wheat grain	14.5	14.5
Wheat bran	15.0	-
Molasses	5.0	5.0
Soybean meal	22.5	25.5
Mineral premix	0.5	0.5
Salt	0.5	0.5
Lime	2.0	2.0
Chemical composition		
DM%	88.4	88.5
CP%	18.1	18.0
ME (M.cal/kg)	2.86	2.77
Fat%	2.7	2.5
NDF%	21.8	28.6
ADF%	12.5	15.3
Ash %	6.5	6.9
Ca%	1.04	1.08
P%	0.61	0.49

**Table 2:** Daily peak ^1^ shed temperature, humidity, THI ^2^, and wind velocity during the experimental period.

	Temperature °C	Humidity %	THI	Wind Velocity km/h
Mean	36.31 ± 3.80	50.98 ± 20.87	80.06 ± 3.42	3.50 ± 2.82
SEM	0.41	2.21	0.37	0.31
*P*-Value (Week)	<0.001	<0.001	-	-

^1^ Recorded daily at 1400 h at two different sites in the shed. ^2^ THI = (0.8 × dry bulb temperature) + (% relative humidity/100) × (dry bulb temperature − 14.4) + 46.4.

**Table 3:** Effect of feeding calf starter and oat hay as TMR or in pelleted form on preweaning performance of crossbred calves (values are least square means).

Variable	Treatment ^1^			SEM	*P*-value	
	CCS	TMR	PTMR		T ^2^	T × W ^3^
Total milk intake kg	384.8	388.4	365.7	11.12	0.12	0.37
Total starter intake kg	22.7	21.1	22.0	0.87	0.74	0.81
Average Starter DMI kg/d	0.28	0.26	0.30	0.01	0.26	0.82
Starter DMI % of BW/d	0.50	0.42	0.52	0.02	0.27	0.86
Initial BW kg	28.2	27.9	26.3	1.00	0.71	
Final BW kg	69.3	69.8	69.2	1.41	0.94	0.23
BW Gain kg	41.1	41.9	42.9	1.32	0.94	0.26
ADG kg/d	0.54	0.55	0.57	0.02	0.45	0.26
Weaning age (d)	76.9	75.3	71.5	1.88	0.07	
Feed efficiency ^4^	0.62	0.63	0.67	0.02	0.57	
Cost per kg gain Pak rupees ^5^	616	575	530	16.69	<0.10	

^1^ Treatments: CCS = conventional calf starter without hay, TMR = total mix ration containing 85% calf starter with 15% chopped OH (chop length 1.5 cm) and PTMR = pelleted form of TMR containing 85% calf starter and 15% OH (pellet size 4 × 18 mm) fed *ad libitum*. ^2^ T = effect of treatment ^3^ T × W = interaction between treatment and week ^4^ Feed efficiency = kg of weight gain/kg of total DMI. ^5^ Cost per kg gain = cost of total starter and milk consumed by the calf till weaning/total weight gained.

**Table 4:** Effect of feeding calf starter and oat hay as TMR or in pelleted form on preweaning body measurement of crossbred calves.

Variable	Treatment ^1^			SEM	*P*-value	
	CCS	TMR	PTMR		T ^2^	T × W ^3^
Withers height, cm						
Initial	71.7	73.0	69.9	0.85	0.36	
Final	84.1	83.6	83.3	0.62	0.16	0.99
Gain	12.4	10.6	13.4	0.84	0.16	0.99
Heart girth, cm						
Initial	67.9	69.3	68.4	0.78	0.80	
Final	80.0	85.0	80.7	1.05	0.07	0.09
Gain	12.1	15.8	12.3	0.89	0.07	0.18
Hip height, cm						
Initial	75.0	70.0	69.6	2.51	0.63	
Final	85.2	81.6	83.2	2.51	0.98	0.73
Gain	10.2	11.6	13.6	0.83	0.98	0.92
Body length, cm						
Initial	65.1	66.4	65.6	1.08	0.90	
Final	80.8	81.8	82.6	0.96	0.19	0.07
Gain	15.7	15.4	17.0	0.89	0.19	0.06

^1^ Treatments: CCS = conventional calf starter without hay, TMR = total mix ration containing 85% calf starter with 15% chopped OH (chop length 1.5 cm) and PTMR = pelleted form of TMR containing 85% calf starter and 15% OH (pellet size 4 × 18 mm) fed *ad libitum*. ^2^ T = effect of treatment ^3^ T × W = interaction between treatment and week.

**Table 5:** Effect of feeding conventional calf starter and oat hay as TMR or in pelleted form on preweaning BUN, BHBA, and glucose concentration of crossbred calves.

Variable	Treatment ^1^			SEM	*P*-value	
	CCS	TMR	PTMR		T ^2^	T × W ^3^
BUN ^4^, mg/dL	11.7	11.3	10.9	0.26	0.5	0.1
BHBA ^5^, mmol/L	0.129	0.138	0.131	0.01	0.72	0.14
Glucose, mg/dL	89.8	85.4	90.1	1.83	0.54	0.34

^1^ Treatments: CCS = conventional calf starter without hay, TMR = total mix ration containing 85% calf starter with 15% chopped OH (chop length 1.5 cm) and PTMR = pelleted form of TMR containing 85% calf starter and 15% OH (pellet size 4 × 18 mm) fed *ad libitum*. ^2^ T = effect of treatment ^3^ T × W = interaction between treatment and week ^4^ BUN = blood urea nitrogen ^5^ BHBA = β-hydroxybutyrate.

**Table 6:** Means of rumen developments measures of sacrificed crossbred male calves fed CCS, TMR, and PTMR.

Variable	Treatment ^1^			SEM	*P*-value
	CCS ^2^	TMR ^3^	PTMR ^4^		
Papillae length (mm)	1.52	1.32	1.53	1.05	0.62
Papillae width (mm)	0.29	0.34	0.32	0.02	0.41
Rumen wall thickness (mm)	1.35	1.67	1.56	0.07	0.75
Papillae concentration (no./cm sq)	103.52	106.83	103.56	1.11	0.15

^1^ Ad libitum fed calf starters ^2^ CCS = Conventional Calf Starter consisting of coarsely ground maize, coarsely wheat grain, Wheat Bran, Soybean Meal 48%, Molasses, Mineral Pre Mix. ^3^ TMR = Total mix ration composing of uniform mixture of coarsely ground Maize, coarsely ground Wheat Grain, Wheat Bran, Soybean Meal 48%, Molasses, Mineral Pre Mix with 15% chopped oat hay. ^4^ PTMR = Pelleted form of above total mix ration composing of uniform mixture of coarsely ground Maize, coarsely ground Wheat Grain, Wheat Bran, Soybean Meal 48%, Molasses, Mineral Pre Mix with 15% chopped oat hay. Pellet size 4 × 18 mm.

**Table 7:** Means of Organ weights with or without ingesta of sacrificed crossbred male calves fed CCS, TMR, and PTMR.

Variable	Treatment ^1^			SEM	*P*-value
	CCS^2^	TMR ^3^	PTMR ^4^		
Body weight with ingesta (kg)	72.10	69.02	67.22	1.88	0.61
Body weight without ingesta	64.66	60.28	60.26	2.12	0.66
EBW % BW	89.33	87.00	90.02	0.92	0.41
Carcass weight (kg)	38.35	35.20	35.07	1.94	0.23
Organ weights with ingesta (kg)					
Total stomach	7.62	7.70	6.97	0.46	0.67
Rumen & Reticulum	5.58	6.31	5.00	0.34	0.69
Omasum	0.77	0.55	0.59	0.07	0.64
Abomasum	1.31	1.30	1.15	0.18	0.79
Intestine	5.00	4.93	4.82	0.21	0.91
Organ weights without ingesta (kg)					
Rumen & Reticulum	1.58	1.18	1.38	0.09	0.31
Rumen weight % of BW without ingesta	2.51	1.96	2.28	0.17	0.21
Omasum	0.44	0.33	0.29	0.03	0.24
Abomasum	0.42	0.35	0.35	0.01	0.13
Abomasum % BW without ingesta	0.66	0.60	0.59	0.02	0.28
Intestine	2.78	2.26	2.82	0.15	0.68
Liver (kg)	1.17	1.12	1.24	0.06	0.60
Spleen	0.51	0.24	0.19	0.10	0.52
Kidney (kg)	0.32	0.27	0.34	0.08	0.17
Heart	0.36	0.32	0.40	0.02	0.20
Lungs	1.32	1.40	0.95	0.17	0.63
Organ length (inches)					
Small intestine	1059.75	982.75	1018.75	37.21	0.24
Large intestine	197.75	203.75	202.75	1.89	0.11

^1^*Ad libitum* fed calf starters ^2^ CCS = Conventional Calf Starter consisting of coarsely ground maize, coarsely wheat grain, Wheat Bran, Soybean Meal 48%, Molasses, Mineral Pre Mix. ^3^ TMR = Total mix ration composing of uniform mixture of coarsely ground Maize, coarsely ground Wheat Grain, Wheat Bran, Soybean Meal 48%, Molasses, Mineral Pre Mix with 15% chopped oat hay. ^4^ PTMR = Pelleted form of above total mix ration composing of uniform mixture of coarsely ground Maize, coarsely ground Wheat Grain, Wheat Bran, Soybean Meal 48%, Molasses, Mineral Pre Mix with 15% chopped oat hay. Pellet size 4 × 18 mm.

**Table 8:** Least square means of health parameters fecal score, respiratory score, and general appearance score of crossbred calves fed conventional calf starter and oat hay as TMR or in pelleted form.

Variable	Treatment ^1^			SEM	*P*-value	
	CCS	TMR	PTMR		T ^2^	T × W ^3^
Fecal Score ^4^	1.25	1.17	1.21	0.04	0.73	0.91
Respiratory Score ^5^	1.13	1.11	1.14	0.02	0.87	0.78
General Appearance Score ^6^	1.13	1.10	1.10	0.02	0.84	0.44

^1^ Treatments: CCS = conventional calf starter without hay, TMR = total mix ration containing 85% calf starter with 15% chopped OH (chop length 1.5 cm) and PTMR = pelleted form of TMR containing 85% calf starter and 15% OH (pellet size 4 × 18 mm) fed *ad libitum*. ^2^ T = effect of treatment ^3^ T × W = interaction between treatment and week ^4^ Fecal score: 1 (normal), 2 (loose), 3 (watery), 4 (mucous, slightly bloody) and 5 (bloody). ^5^ Respiratory score: 1 (normal), 2 (slight cough), 3 (moderate cough), 4 (moderate to severe cough), and 5 (severe cough). ^6^ General appearance score: 1 (normal and alert), 2 (drooping of ears), 3 (head and ears dropped), 4 (dull, lethargic), and 5 (severely lethargic).
